# Through the Looking Glass: Visualizing Leukemia Growth, Migration, and Engraftment Using Fluorescent Transgenic Zebrafish

**DOI:** 10.1155/2012/478164

**Published:** 2012-07-08

**Authors:** Finola E. Moore, David M. Langenau

**Affiliations:** ^1^Department of Pathology and Cancer Center, Massachusetts General Hospital, Building 149, Charlestown, MA 02129, USA; ^2^Harvard Stem Cell Institute, Holyoke Center, Suite 727W, 1350 Massachusetts Avenue, Cambridge, MA 02138, USA; ^3^Department of Genetics, Harvard Medical School, 77 Avenue Louis Pasteur, NRB 0330, Boston, MA 02115, USA

## Abstract

Zebrafish have emerged as a powerful model of development and cancer. Human, mouse, and zebrafish malignancies exhibit striking histopathologic and molecular similarities, underscoring the remarkable conservation of genetic pathways required to induce cancer. Zebrafish are uniquely suited for large-scale studies in which hundreds of animals can be used to investigate cancer processes. Moreover, zebrafish are small in size, optically clear during development, and amenable to genetic manipulation. Facile transgenic approaches and new technologies in gene inactivation have provided much needed genomic resources to interrogate the function of specific oncogenic and tumor suppressor pathways in cancer. This manuscript focuses on the unique attribute of labeling leukemia cells with fluorescent proteins and directly visualizing cancer processes *in vivo* including tumor growth, dissemination, and intravasation into the vasculature. We will also discuss the use of fluorescent transgenic approaches and cell transplantation to assess leukemia-propagating cell frequency and response to chemotherapy.

## 1. Zebrafish Models of Leukemia 

Zebrafish models of hematological malignancies exhibit striking similarities with human and mouse disease [1–7], yet afford unique avenues of study due to imaging modalities that permit direct visualization of fluorescently labeled blood cells within live animals. As with mouse and human disease, zebrafish leukemias are distinguished from lymphomas by the infiltration of leukemic cells into the marrow. Lymphomas are predominantly located as masses throughout the body, including lymph nodes in mouse and human, and have no or little infiltration into the marrow [8]. Leukemias are also classified as acute or chronic. Acute leukemias are arrested at early stages of maturation, are highly proliferative, and advance quickly in patients [8]. By contrast, chronic leukemias are arrested at later stages of maturation and resemble functional, yet abnormal, blood cell counterparts. Although characterized by increased circulating white blood counts, chronic leukemias are often much slower growing and take months or years to progress. Leukemias can be further subdivided based on the blood lineage in which cells have become transformed [8]. To date, zebrafish models of Acute Lymphoblastic Leukemias (ALL), Acute Myeloid Leukemia (AML), and Myeloproliferative Neoplasms (MPN) have been described.

Zebrafish first emerged as a powerful genetic model of leukemia with the description of transgenic approaches in which cMYC was overexpressed in developing thymocytes [7]. Utilizing the *rag2* promoter to drive both MYC and GFP expression, transgenic zebrafish T-cell acute lymphoblastic leukemias (T-ALLs) could be easily visualized in live animals. In this model, fluorescently labeled T cell precursors resident in the thymus were the T-ALL-initiating cell type and disseminated widely over the course of tumor progression [7]. Moreover, GFP+ thymocytes exhibited stereotypical homing to the nasal placode, periocular space, and kidney marrow when assessed by serial fluorescent imaging over days [7]. Subsequent studies developed conditional approaches to create fluorescent transgenic zebrafish models of T-ALL that utilized CRE-Lox or tamoxifen-inducible MYC-ER strategies [5, 9]. Interestingly, withdrawal of tamoxifen and subsequent inactivation of MYC expression led to regression of fluorescently labeled T-ALL; however, leukemia regression was not observed in *pten* mutant fish or those that overexpressed activated *Akt* [9]. These data indicate that *Akt* pathway activation is sufficient for tumor maintenance in this model. Additional studies have utilized fluorescence imaging to assess synergy between MYC and Bcl2 [5, 10] and NOTCH1-ICD [1]. Moreover, human *NOTCH1-intracellular domain-EGFP* transgene expression induces fluorescently labeled T-ALL with a long latency of >6 months in mosaic and stable transgenic zebrafish [6]. Finally, forward genetics screens that utilize ENU (N-Ethyl-N-nitrosourea-) induced mutagenesis are easily performed in zebrafish due to their large clutch size and accessible observation of phenotypes. Utilizing this approach, the Trede group mutagenized *Tg(lck:GFP)* transgenic fish and visualized animals for fluorescently labeled T-ALL onset in F1 and F2 animals, identifying both dominant and recessive mutations that affect T-ALL onset [11]. Mapping of mutations that are found in these mutant lines will likely uncover novel mechanisms that drive T-ALL onset and growth in both zebrafish and man.

Many exciting new models of hematopoietic malignancy have been created including B-cell acute lymphoblastic leukemia (B-ALL), acute myeloid leukemia (AML), and myeloproliferative neoplasm (MPN). For example, Sabaawy et al. developed a model of B-ALL by overexpressing *EGFP-TEL-AML1* from a ubiquitous transgene promoter. In this model, 16 of 545 transgenic animals developed B-ALL by 8–12 months of age [2]. Zhuravleva et al. generated transgenic zebrafish in which the *MYST3/NCOA2* fusion gene was expressed under control of the *spi1 *promoter [12]. 2 of 180 *MYST3/NCOA2-EGFP* mosaic transgenic animals developed AML at 14 and 26 months. Two models of MPN have also been developed. Le et al. utilized CRE/Lox techniques to conditionally activate *kRASG12D* in developing embryos [3]. A subset of these animals went on to develop myeloproliferative neoplasm with a latency of 66.2 ± 23.1 days (*n* = 10  of 19 fish). Forrester et al. also developed a conditional CRE/Lox transgenic approach to model MPN [13]. Specifically, *NUP98-HOXA9* was conditionally activated in *pu.1* expressing cells, leading to 23% of adult *NUP98-HOXA9*-transgenic fish developing MPN by 19–23 months of age. Finally, several investigators have utilized heat-shock transgenic approaches to uncover early developmental effects of fusion oncogenes in blood development, including *AML1-ETO, RUNX1-CBF2T1,* and *TEL-JAK2* [4, 14, 15]. These heat-shock approaches drive transgene expression during early development and often result in aberrant arrest of cells in early stages of blood development. However, the development of frank leukemia in heat-shock inducible transgenic lines has yet to be reported. Taken together, zebrafish have fast emerged as a novel animal model of leukemia and are poised to contribute to our understanding of the molecular pathogenesis of human disease.

## 2. Fluorescent Transgenic Approaches to Label ****Leukemia Cells

Many studies have employed the use of stable transgenic zebrafish to drive oncogenic transgene expression in a tissue-specific manner including pancreatic adenocarcinoma [16], hepatocellular carcinoma [17], melanoma [18–20], embryonal rhabdomyosarcoma [21], and leukemia. By and large, investigators have used oncogene fusions with GFP to create tumors that are fluorescently labeled. For example, we and others have generated *EGFP-Myc, NOTCH1-GFP, EGFP-TEL-AML1*, and *MYST3/NCOA2-EGFP* fusions to drive leukemogenesis while also fluorescently labeling leukemic cells [2, 6, 7, 12]. Although these approaches have been largely successful in generating fluorescently labeled leukemias, it is worth noting that fluorescent protein expression is linked with oncogene localization within the cell and protein stability. For example, MYC is a nuclear transcription factor with a half-life of ~30 minutes in non-transformed cells. Thus, the EGFP-MYC fusion protein is rapidly turned over in normal thymocytes prior to GFP maturation into a functional fluorescent molecule, precluding the use of fluorescence to identify stable transgenic *Tg(rag2:EGFP-Myc)* animals at 5 days of life. However, the *EGFP-Myc* transgene is stabilized following transformation leading to weak, nuclear fluorescent protein expression in T-ALL. Fluorescent protein fusions can also exhibit reduced transforming activity depending on cellular context. For example, we have developed a zebrafish model of *kRASG12D*-induced embryonal rhabdomyosarcoma but have been unable to model this disease using the same transgene promoter to drive expression of a GFP fusion with kRASG12D. By contrast, others have used similar RAS fusion constructs to generate fluorescently labeled hepatocellular carcinoma, pancreatic adenocarcinoma, and melanoma [16, 17, 19, 20]. To obviate issues surrounding the function of fluorescent protein-oncogene fusions, it is possible to utilize dual transgenic approaches to drive both the oncogene and fluorescent protein within the same cell types. For example, *Tg(rag2:Myc)* lines could be bred to *Tg(rag2:GFP)* fish. The resulting progeny would develop T-ALL that expresses high fluorescent protein expression. 

**Figure 1:**
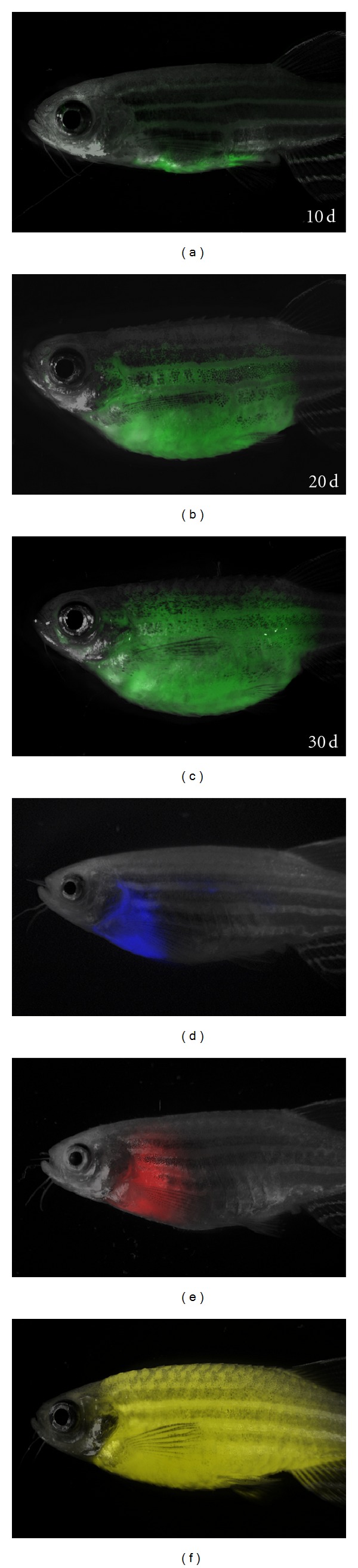
Fluorescently labeled Myc-induced T-ALLs from CG1-strain zebrafish engraft into nonirradiated CG1-strain recipients. (a)–(c) GFP-labeled T-ALLs were isolated from primary leukemic fish, and 1 × 10^3^ FACS sorted GFP-labeled leukemia cells were transplanted into nonirradiated CG1-strain animals and scored for engraftment at 10, 20, and 30 days posttransplantation. (d)–(f) T-ALL transplant recipients that express Amcyan (d), dsRED (e), and zsYellow (f) under the *rag2* promoter. Panels are merged images of fluorescent and brightfield photographs. Images were originally published in [23].

Although stable transgenic zebrafish have been used to develop robust models of cancer, mosaic transgenic approaches provide many unique benefits for modeling cancer in zebrafish. First, stable transgenic zebrafish are often prone to developing early onset cancers, making maintenance of stable lines difficult. Second, the creation of stable transgenic zebrafish is time-consuming and requires crossing putative transgenic animals to identify founder fish. Although the transgenesis with Tol2 transposase has facilitated the creation of stable transgenic lines, complex breeding strategies are required to introduce additional transgenes and/or mutant alleles into a given background. Such approaches often require multiple generations to develop strains of interest. By contrast, mosaic transgenesis relies on the ability of multiple, linearized transgenes to incorporate into the genome as concatamers when microinjected into one-cell stage zebrafish, ultimately culminating in the coexpression of transgenes in developing disease. We have successfully used this approach to show that *kRASG12D* collaborates with *p53 *loss to induce early onset embryonal rhabdomyosarcoma [22] and work from Feng et al., elegantly showed that mosaic transgenesis can be used to modify *Myc*-induced T-ALL through coinjection of activated *Akt* [10]. We have used similar approaches to develop T-ALLs that coexpress MYC and various fluorescent reporters including AmCyan, GFP, zsYellow, dsREDexpress, and mCherry [23, 24]. In these experiments, embryos are coinjected with *Myc* and fluorescent protein under transcriptional control of the *rag2* promoter. A small cohort of animals develop fluorescently labeled thymi that eventually progresses into T-ALL. Using this approach, we have been able to create T-ALLs in various genetic backgrounds, permitting the creation of syngeneic strain fish that develop multicolored T-ALL (Figure 1) [23]. Finally, we have recently utilized mosaic transgenesis to coexpress Notch1a-ICD, MYC, and GFP by coinjection of three transgenes simultaneously into one-cell stage animals [1]. In summary, while some fluorescent transgenic approaches can be limited by fusion stability, early onset of cancer, and genetic background, other fluorescent transgenic approaches have been able to overcome these limitations. Such approaches provide rapid assays to identify collaborating oncogenic/tumor suppressor pathways in leukemia.

## 3. Cell Transplantation Approaches to Visualize Tumor Cell Engraftment 

Investigators have utilized cell transplantation of fluorescently labeled cancer cells into sublethally irradiated adult zebrafish to assess tumorigenicity [7]. For example, Traver et al. optimized cell transplantation of both blood and leukemic cells into gamma-irradiated animals [7, 25]. Specifically, recipient fish were irradiated with 20–25 Gy two days prior to cell transplantation and then injected with fluorescently labeled donor cells into the peritoneal cavity or sinus venosis. For T-ALL, animals can be injected with 1 × 10^6^ cells and assessed for fluorescently labeled leukemia engraftment at 10 days posttransplantation [7, 25]. Imaging of engraftment can be further facilitated by transplantation into optically clear strains of zebrafish that lack iridiphores and melanocytes—aptly named *casper* [26]. *Casper* fish were created by breeding together *roy* and *nacre* mutants and must be maintained as double homozygous mutant animals. These fish are transparent as adults, facilitating detailed imaging of cell migration, metastasis, and kinetics of tumor growth. For example, recent work has shown that blood cells can be tracked and counted within the circulation of live adult fish using an integrated optical system that combines a laser scanning confocal microscope and an *in vivo* flow cytometer [27]. 

Although transplantation of donor cells into irradiated recipients is a powerful tool to assess short-term engraftment potential, long-term engraftment of cells >20 days posttransplantation is often not possible due to the recovery of the host immune system and subsequent attack of engrafted cells [23, 28]. To avoid immune rejection, Mizgirev and Revskoy recently developed syngeneic zebrafish strains and created robust models of transplantable, chemically induced hepatocellular carcinomas, hepatoblastomas, cholangiocarcinoma, and pancreatic carcinoma [29–31]. Specifically, syngeneic zebrafish were created by fertilizing eggs with UV-inactivated sperm, then subjecting eggs to heat-shock [29]. Female gynogenic diploid animals were raised to adulthood and the process repeated. The resulting progeny were genetically similar and could be maintained by incrossing or mating male fish back to the founding mother. Several lines were created using this method including clonal golden strain 1 and 2 (CG1 and CG2). Adoptive transfer of chemical-induced cancers and *Tg(rag2:EGFP-Myc-)* induced T-ALLs from CG2-strain fish could engraft disease into syngeneic recipients [31]. Moreover, fluorescently labeled rhabdomyosarcoma and T-ALL cells arising in CG1 strain fish could also engraft into nonirradiated, recipient fish [23, 24]. Taken together, these results illustrate the power of cell transplantation and use of syngeneic zebrafish to study leukemia cell engraftment.

## 4. Cell Transplantation Approaches to Examine Tumor Cell Homing and Intravasation into Vessels 

Blood cells and their dynamic cell movements can be easily visualized in live fluorescent transgenic zebrafish. For example, researchers have tracked the migration of various blood lineages including erythroid and macrophage progenitors [25, 32–34]. Importantly, hematopoietic stem cell (HSC) movement can also be followed in *Tg(CD41:eGFP)*, *Tg(cmyb:GFP)*, *Tg(runx1:GFP),* and *Tg(lmo2:GFP)* transgenic zebrafish larvae [35–40]. Moreover, fluorescently labeled blood cells can also be tracked in adult fish [27, 41]. Capitalizing on cell transplantation approaches, investigators have also utilized fluorescence imaging to visualize normal hematopoietic cell homing in live animals. For example, Bertrand et al. visualized HSC homing to the caudal hematopoietic tissue by transplanting *Tg(CD41:eGFP; gata1:dsRed)* cells into irradiated recipients [36]. We have also described the homing of *Tg(lck:GFP)*+ T cells back to the thymus following transplantation of cells into larval wildtype fish [42]. While malignant GFP+ T-ALL lymphoblasts also migrate to the thymus, they exhibit robust and specific homing to the olfactory bulb [6, 7]. These studies demonstrate the ease of visualizing cell migration and homing to specific anatomically defined sites within live animals using fluorescently labeled normal hematopoietic and leukemic cells. 

**Figure 2:**
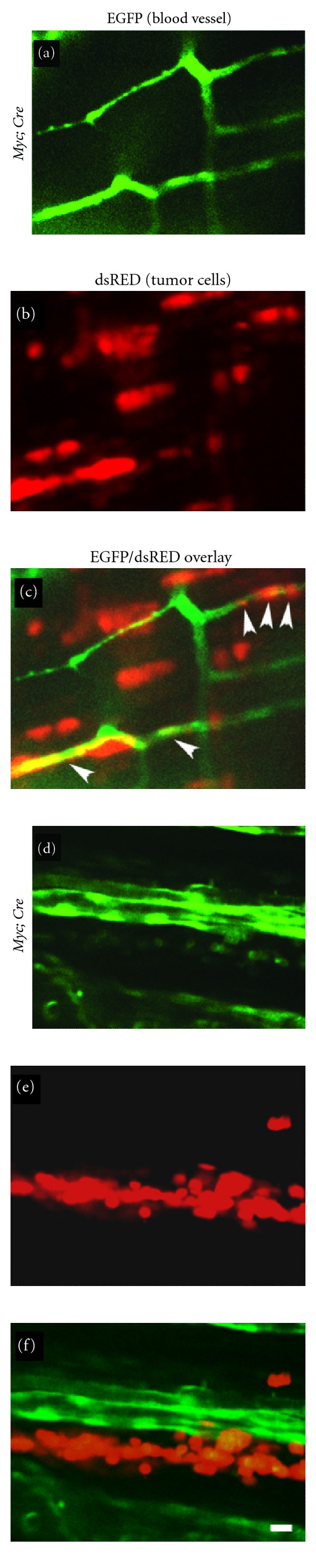
Zebrafish T-lymphoblasts overexpressing bcl2 spread locally but fail to intravasate into vasculature. (a)–(c) dsRED2-expressing lymphoma cells (b) from the *Myc; Cre* fish intravasate into EGFP-labeled vasculature (a) of the transplant host *Tg(fli1:EGFP); Casper* by 6 days posttransplantation (see arrowheads in (c)). (d)–(f) In contrast, dsRED2-expressing lymphoma cells (e) from the *Myc; Cre; bcl2* fish fail to intravasate vasculature (d) of the transplant hosts by 6 days posttransplantation (compare (f) with (c)). Note aggregates of the *Myc; Cre; bcl2* lymphoma cells in (e) and (f). Scale bar is 10 *μ*m. Reprinted from [10].

Intravasation of cancer cells into the vasculature is a critical step in cancer progression, allowing the spread of tumor cells beyond the site of origin [43]. The extent to which lymphoblasts disseminate is the clinically defining characteristic of T-lymphoblastic lymphoma (T-LBL) and acute T-lymphoblastic leukemia (T-ALL) [8]. In T-LBL, transformed lymphoblasts are confined to mediastinal masses, while frank leukemia involves dissemination of cells to the marrow. Remarkably, this disease transition was recently visualized in zebrafish transplanted with fluorescently labeled lymphoblasts [10]. For example, RFP+ lymphoblasts from *Myc*-induced T-ALL were able to intravasate into *Tg(fli:GFP)*-labeled vasculature, while cells that overexpressed the antiapoptotic protein Bcl2 were unable to enter the vasculature and, thus, were arrested in a T-LBL state (Figure 2) [10]. Remarkably, treatment of transgenic zebrafish that overexpressed MYC and Bcl2 with an antagonist to Sphingosine-1-Phosphate (S1P1), a T-cell adhesion and migration protein, promoted invasion into the vasculature [10]. These elegant studies by Feng et al. were the first to directly visualize the molecular mechanisms governing the transition of T-LBL to T-ALL and underscore the power of imaging dynamic cellular processes in fluorescently labeled animals.

## 5. Fluorescence Imaging to Visualize Leukemia Responses to Drug Treatment and ****Gamma-Irradiation

**Figure 3:**
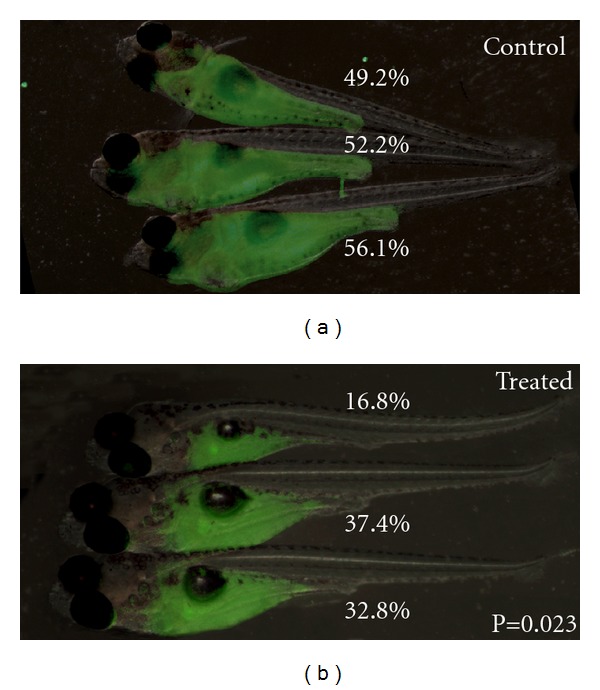
Syngeneic zebrafish transplant models of T-ALL are a powerful tool for drug discovery: T-ALL growth is suppressed by cyclophosphamide treatment. Approximately 200 cells/5 nL were engrafted into 5-day-old syngeneic CG2 * *larvae. Engrafted animals were treated with cyclophosphamide (400 mg/L dissolved in fish water) beginning 5 days posttransplantation. Images of control (a) and treated animals. (b) Tumor growth was assessed based on the percentage of body taken over by GFP+ T-ALL and compared using  *t*-test calculations. This work was performed in [31] and later published in [61].

Fluorescence imaging of transplanted cancer cells can also be used to visualize response to chemotherapy and radiation. For example, the Revskoy group recently showed that GFP-labeled T-ALL cells could be serially transplanted into syngeneic strain larvae [31]. Treatment of transplant recipients with vincristine or cyclophosphamide reduced tumor burden (Figure 3) and extended lifespan significantly [31]. These experiments established that high-throughput cell transplantation assays can generate large cohorts of animals for drug screens and showed that zebrafish T-ALL responds to the same drugs that are used to treat human T-ALL patients [31]. In addition, fluorescently labeled cells can be assessed for response to radiation. For example, we have shown that engrafted GFP-labeled T-ALLs that coexpress *EGFP-bcl2* and the *Myc *transgene failed to undergo apoptosis following 20 Gy of gamma-irradiation [44]; however, T-ALLs that express only *Myc *were ablated by 4 days postirradiation, suggesting that *Myc*-induced T-ALL have an intact *p53 *DNA damage pathway. 

## 6. Cell Transplantation Approaches to Quantify Leukemia Propagating Cell Frequency and Aggression

Leukemia-propagating cells (LPCs) have the capacity to produce all the other cell types contained within the leukemia, are responsible for continued tumor growth, and ultimately drive relapse. Investigators have used fluorescence-activated cell sorting (FACS) to identify unique cell populations and limiting dilution cell transplantation to assess if molecularly defined leukemia cells retain LPC activity in human disease. For example, in AML a rare CD34+, CD38− cell enriches for leukemia-propagating potential [45, 46]. In T-ALL, it has been suggested that CD34+ CD7+ cell populations are enriched in LPCs [47]. Despite enormous efforts aimed at defining if and what cell surface markers define LPC activity, relatively little is known about the molecular mechanisms that drive leukemia propagating activity. For example, elegant work from Jean Soulier's group has shown xenograft transplantation of primary human T-ALL into immune-compromised mice selected for a small subset of clones found within the diagnosis leukemia [48]. These clones contained specific genomic lesions that likely increase leukemia aggression and increase the frequency of LPCs within the bulk of the leukemia mass [48]. Yet, despite the identification of recurrent genomic changes associated within continued clonal evolution, the mechanisms driving these relapse-associated processes are largely unknown.

 The process by which leukemic cells acquire mutations to increase aggression and frequency of LPCs has been difficult to study in human and mouse models of disease. However, recent work from the Trede group has utilized serially passaged fluorescently labeled zebrafish T-ALLs to demonstrate that leukemias become more aggressive and develop with shortened latency [49]. To assess genetic changes acquired between the primary and evolved clones, array comparative hybridization studies were completed to identify recurrent genomic DNA alterations associated with increased aggression. An average of 34 new copy number aberrations (CNAs) were identified in T-ALLs following serial passaging, a majority of which were also found in human T-ALL [49]. Clonal evolution can also result in increased numbers of LPCs contained within the leukemia mass [48]. To directly assess LPC frequency within the bulk of the tumor mass, we have pioneered high-throughput limiting dilution cell transplantation approaches and showed that 1% of Myc-induced T-ALL cells has the capacity to remake leukemia in syngeneic recipient animals [23, 24]. Following serial passaging, a subset of clones can increase LPC activity with up to 16% of cells now capable of inducing leukemia in transplant recipient animals [23]. Similar array CGH studies as described by Rudner et al. [49] are currently underway to identify recurrent CNAs associated with modulating LPC frequency in zebrafish T-ALL. Taken together, we believe that unbiased genetic approaches, when coupled with limiting dilution cell transplantation assays in zebrafish, will likely uncover the mechanisms driving relapse-associated changes in aggression and LPC frequency in human disease.

## 7. Conclusion and Challenges for the Future

Zebrafish has fast emerged as a powerful model of leukemia. When coupled with fluorescent transgenic approaches and powerful imaging techniques, these models are uniquely positioned to uncover mechanisms driving tumor dissemination, progression, and relapse. Moreover, the use of multifluorescent transgenic animals will allow for labeling of tumor cell compartments similar to those defined in RAS-induced rhabdomyosarcoma models [21, 50] and for the visualizing of leukemia growth in relation to supportive cell types including vasculature, fibroblasts, and macrophages. Moreover, though not the focus of this paper, cell transplantation approaches that utilize fluorescently labeled, human leukemia cells into either zebrafish embryos or adults will likely provide novel experimental models to assess tumor growth and response to therapy [51–60], capitalizing on the numbers of disease animals that can be created by microinjection and direct visualization of tumor growth *in vivo.*
